# Uncertainty Estimation in Predicting River Discharge Using Probabilistic Machine Learning and Conformal Prediction

**DOI:** 10.3390/s26185800

**Published:** 2026-09-13

**Authors:** Erfan Abdi, Mohammad Taghi Sattari, Mahesh Pal, Adam Milewski, Halit Apaydin

**Affiliations:** 1 Department of Water Engineering, Faculty of Agriculture, University of Tabriz, Tabriz 51666-16471, Iran; erabdi78@gmail.com; 2 Water Sciences and Hydroinformatics Research Center, Khazar University, Mahsati Str. 41, AZ 1096 Baku, Azerbaijan; apaydin@ankara.edu.tr; 3 Department of Agricultural Engineering, Ankara University, Ankara 06100, Türkiye; 4 Department of Civil Engineering, National Institute of Technology, Kurukshetra 136119, India; mahesh.pal@nitkkr.ac.in; 5 Department of Geology, University of Georgia, 210 Field Street, Athens, GA 30602, USA

**Keywords:** probabilistic gradient boosting machines, natural gradient boosting, conformal prediction, uncertainty, effective coverage, predicted interval width

## Abstract

Reliable streamflow forecasting with quantified uncertainty is essential for water resource management, flood mitigation, and climate adaptation in semi-arid regions. This research introduces a framework that combines three conformal prediction techniques, including Split Conformal Prediction (SplitCP), Cross Validation Plus (CV+), and conformal quantile regression, with two probabilistic machine learning algorithms, namely Natural Gradient Boosting (NGBoost) and Probabilistic Gradient Boosting Machines (PGBM), to quantify uncertainty in hydrological modeling of the Sattarkhan Dam in East Azerbaijan Province, located in north-eastern Iran. The data collected ranged from 21 March 1996 to 22 September 2022 and were divided into two chronological groups: training (70%) and testing (30%) for modeling. Probabilistic prediction quality was evaluated using the continuous ranked probability score (CRPS) and negative log-likelihood (NLL). In contrast, for conformal prediction, we used the mean predicted interval width, effective coverage, and coverage width criteria. Results in terms of correlation coefficient (CC), mean absolute error (MAE), and root mean square error (RMSE) with the test dataset suggest improved performance by NGBoost (RMSE: 0.833 m^3^/s, CC: 0.918, MAE: 0.375) using optimal values of user-defined parameters in comparison to PGBM (RMSE: 0.909 m^3^/s, CC: 0.902, MAE: 0.388). NGBoost outperforms PGBM in probabilistic prediction. Its higher coverage indicates CV+ as the most effective uncertainty estimation method for this dataset. These findings support model reliability and inform future decision-making. These findings support operational forecasting and risk-informed decision-making in semi-arid regions. Also, the framework provides a transferable template for similar hydrological uncertainty studies.

## 1. Introduction

Reliable and precise predictions of streamflow are essential for effective water resource management, which is important for assessing rural water needs and allocating water resources among competing uses, forecasting floods and droughts, generating hydropower, and sustaining ecological habitat. Streamflow is the product of complex hydrological processes, characterized by nonlinearity, temporal correlation, and spatial variability arising from interactions between meteorological inputs and catchment measurements [1,2,3]. For years, hydrologists have attempted to model these processes to produce timely predictions from one minute for flash floods or one season for reservoir operations. In other words, accurate streamflow predictions are important for flood forecasting, water resource planning, hydropower scheduling, and ecosystem management. In contrast to past decades of hydrological modeling, data availability (both historical and near-real-time) and machine learning (ML) methods provide a powerful new platform for learning complex, nonlinear relationships between meteorological inputs and space–time variability in river discharge. A wide range of machine learning approaches, from tree-based models (decision tree, random forest) through neural networks, support vector machines, and deep neural networks, have been applied to predict river flow in many catchments [4,5,6,7,8,9]. The modeling appears to outperform empirical modeling techniques with the right training datasets and appropriate input pre-processing [10,11].

Although data-driven models perform well, they can be overly optimistic and lack adequate communication of predictive uncertainty that would inform a risk-aware decision-making process [12,13,14]. Thus, appropriate uncertainty estimation methods are needed to provide actionable streamflow forecasts, particularly during extreme events and for ungauged basins [15,16,17]. The importance of uncertainty quantification lies in its direct impact on flood safety, water allocation, and infrastructure planning; overconfident predictions during floods can delay evacuations and cause catastrophic damage, while poorly bounded forecasts during droughts can lead to unsustainable water extraction and economic losses. In ungauged basins or under changing climate conditions, where observational data are limited and historical relationships may shift, uncertainty estimates become essential for understanding prediction reliability and guiding adaptive management strategies [18]. Zhang et al. [19] evaluated eight data-driven models and five preprocessing methodologies for short-term streamflow forecasting in four stations in the East River basin (China). All methods used included multiple linear regression (MLR), artificial neural networks (ANN), and wavelet decomposition (WD), and the wavelet–ANN (W-ANN) method was used for 1-month-ahead forecasts at Longchuan station. Fan et al. [20] proposed an explainable machine learning model with uncertainty quantification (UQ) to improve multi-step reservoir inflow forecasting, addressing the limitations of traditional ML methods, namely poor multi-day forecast accuracy, lack of explainability, and absence of UQ. To overcome these challenges, an encoder–decoder long short-term memory (ED-LSTM) network for multi-step forecasting is employed; the SHapley Additive exPlanation (SHAP) technique is used to interpret the influence of hydrometeorological factors, and a novel UQ method is developed to assess prediction trustworthiness. The proposed framework was applied to forecast 7-day inflow in both snow-dominant and rain-driven reservoirs. Results demonstrate that ED-LSTM achieves high forecasting accuracy for short lead times, while the UQ method provides reliable uncertainty estimates, covering 90% of observations at a 90% confidence level. Lin et al. [21] used mapping-bias-learning models to address data-driven streamflow prediction bias arising from data completeness and environmental variability. Two experimental groups were used in the studies: one in the Andun River basin (China) and one across 273 watersheds in CONUS. Sixteen mapping-bias-learning models and four mapping-learning-alone models were derived from three machine-learning algorithms and one time-series method. Özdoğan-Sarıkoç and Dadaser-Celik [22] compared the performance of a data-driven model (NARX) and a physically based model (SWAT) for predicting reservoir volumes and streamflow at the semi-arid Tersakan Basin, Türkiye. Calibration and validation were conducted for both models at the Ladik and Yedikir reservoirs and at the basin outlet. Overall, the NARX model showed improved performance for all prediction tasks compared to SWAT, especially in data-scarce conditions. Overall, both calibration and validation performance were best for the Ladik reservoir, and although performance declined for both models at the basin outlet, the Ladik reservoir showed the best overall performance. The results indicate that, especially when input data are scarce or uncertain, data-driven approaches such as NARX show promise as viable alternatives to physically based models. Jia et al. [23] address limitations in probabilistic runoff forecasting using Mixture Density Networks (MDN), specifically bias from distributional misspecification and overly wide prediction intervals that reduce practical utility. To overcome these issues, innovatively integrated the Weighted Conformal Inference (WCI) strategy, which accounts for distributional shifts in runoff sequences, with MDN to develop the WCI-MDN model for runoff interval prediction. Six models were constructed and evaluated using data from 222 basins in the CAMELS-AUS (Catchment Attributes and Meteorology for Large-Sample Studies, Australia) dataset: MDNs and WCI-MDNs under three distributions, Gaussian Mixture Model (GMM), Laplace Mixture Model (LMM), and Countable Mixtures of Asymmetric Laplacians (CMAL). Results showed that among MDNs, the LMM distribution performed best, followed by CMAL and GMM.

Uncertainty in machine learning predictions refers to the confidence associated with a model’s output. Usually, uncertainty is determined through probabilistic prediction rather than point prediction [24]. Probabilistic prediction provides a range of predictions, usually defined by mean and standard deviation for each sample. A higher standard deviation indicates greater uncertainty in predictions. Uncertainty estimation in the predictions of various machine learning models can be helpful in their deployment in real-world problems. Beyond accurate predictions from different machine learning models, the availability of reliable uncertainty estimates can help them be applied to real-world problems in automated decision-making. Several approaches, including quantile regression, Monte Carlo Dropout, ensemble methods, and conformal prediction-based uncertainty estimation, are proposed in the literature [25].

Accurate river discharge forecasts are critical to water resource management, flood mitigation, and infrastructure development around important structures such as dams. In the absence of uncertainty quantification, uncertainty can lead to over- or underestimating flow, resulting in mismanagement of water resources, increased risk of flooding, and/or structural failures. Probabilistic machine learning models provide forecasts with uncertainty bounds, thereby presenting a broader view of possible results than point estimates. This is particularly beneficial in more complex and variable environments, like the Sattarkhan Dam catchment, where climate and hydrology have considerable variability.

Given the importance and potential of probabilistic machine learning models and uncertainty estimation based on adaptive prediction, the present study investigates the effectiveness of probabilistic machine learning algorithms for river discharge prediction in the semi-arid Sattarkhan Dam catchment. The specific objectives of this study are: (1) To evaluate and compare the predictive performance of two state-of-the-art probabilistic gradient boosting methods, Natural Gradient Boosting (NGBoost) and Probabilistic Gradient Boosting Machines (PGBM), for daily streamflow forecasting; (2) to quantify the predictive uncertainty associated with each model using three conformal prediction techniques, namely Split Conformal Prediction (SplitCP), Cross Validation Plus (CV+), and conformal quantile regression; (3) to identify the most effective combination of probabilistic model and conformal prediction method for generating well-calibrated prediction intervals in a semi-arid hydrological setting; and (4) to assess the reliability of the probabilistic forecasts using proper scoring rules (Continuous Ranked Probability Score and Negative Log-Likelihood) and uncertainty metrics (sharpness, effective coverage, and prediction interval width). The methodological framework developed herein, comprising PACF-based lag selection, Optuna-driven hyperparameter optimization, probabilistic gradient boosting, and conformal prediction, is designed to be transferable and provides a template for future applications to diverse hydrological regimes, including humid, snow-dominated, and large river systems.

## 2. Dataset and Methodology

### 2.1. Study Area

**Figure 1:**
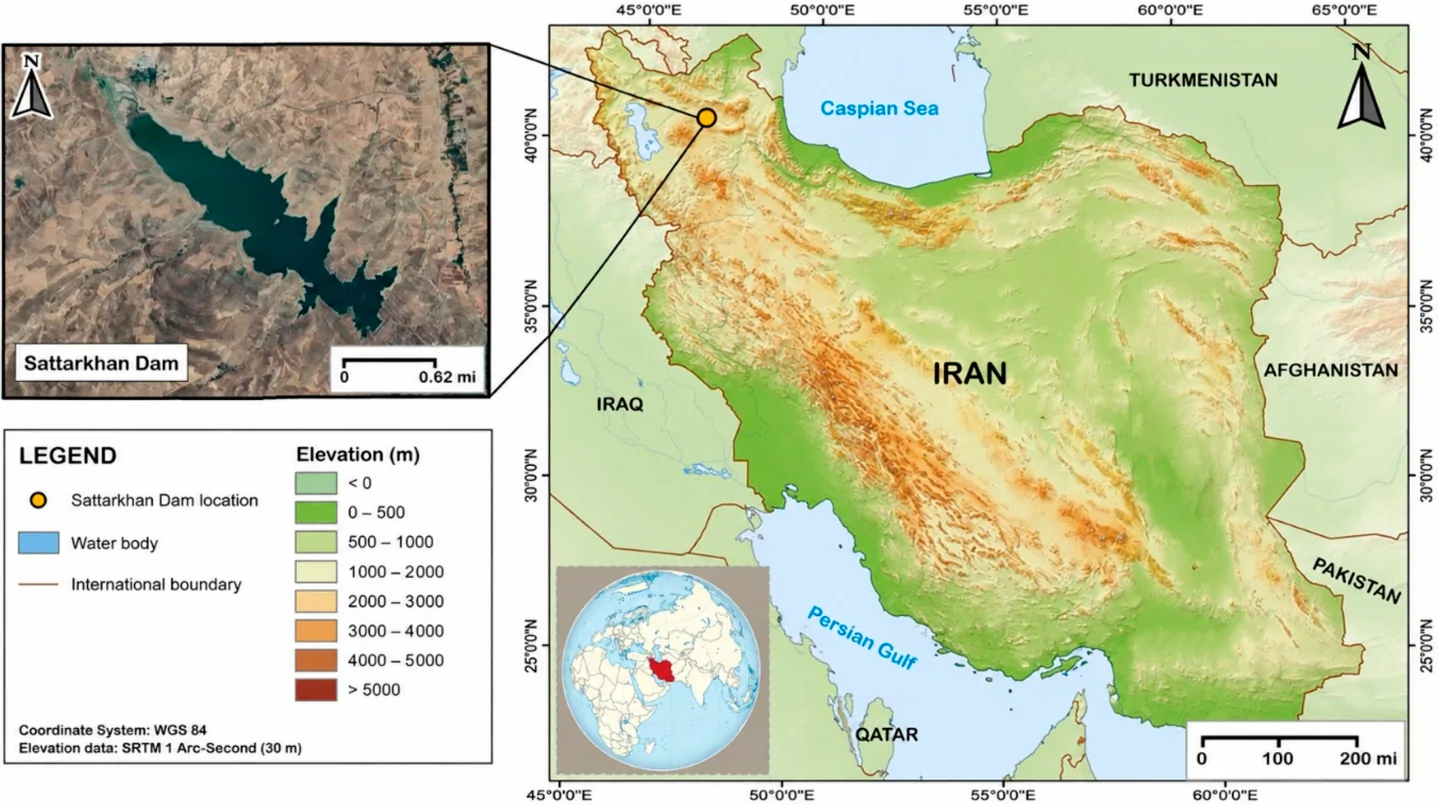
Location of the Sattarkhan Dam in Iran.

The coordinates of Sattarkhan Dam, lying in East Azerbaijan, north-eastern Iran, are 38.48° N latitude and 47.07° E longitude (Figure 1). These coordinates place the dam approximately 15 km west of the city of Ahar, in the mountainous region of northwestern Iran. The dam is constructed with an impermeable clay core, is approximately 75 m tall, and extends approximately 350 m across its crest. The dam’s reservoir can store 131 million cubic meters of water [26]. The dam serves to provide flood control, to supply water to its residents, and to irrigate around 12,000 hectares of farmland. A significant aspect of the dam infrastructure is a diversion tunnel approximately 5.5 m in diameter that provides water intake and discharge, in addition to flood control. The dam is located in a catchment area of approximately 950 km^2^, and the area has a semi-arid, cold climate typical of East Azerbaijan. The area experiences relatively cold winters and moderate precipitation, which is approximately 297 mm per year. The elevation near the dam, close to the city of Ahar, is approximately 1377 m above sea level, and the reservoir’s maximum elevation is approximately 1454 m.

### 2.2. Datasets

**Figure 2:**
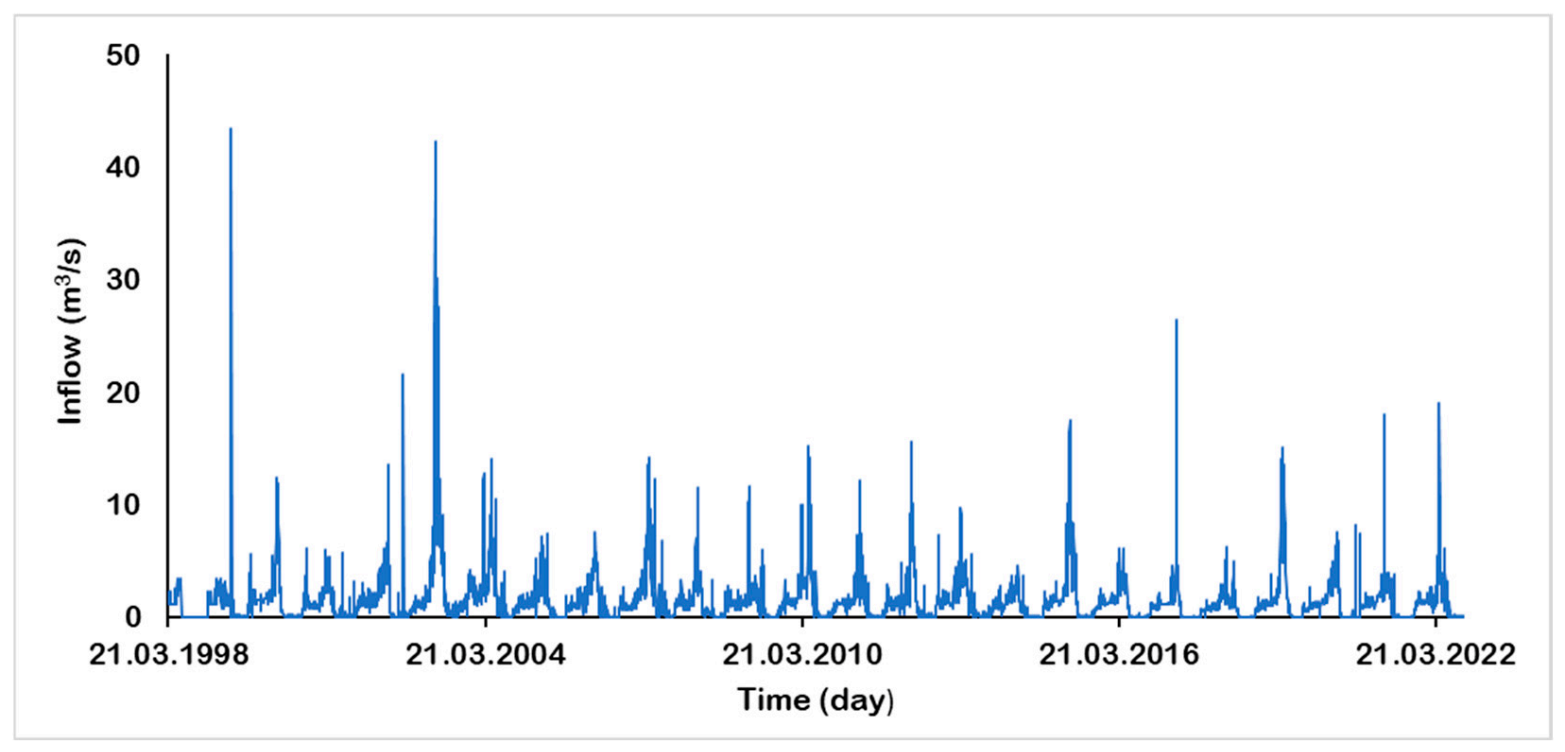
Time series of Sattarkhan Dam the reservoir inflow for 25 years.

The data, covering the period from 21 March 1996 to 22 September 2022, were obtained from the regional water authority. Missing values were managed using arithmetic mean interpolation to preserve the data. The data were then rescaled using Z-score normalization, which sets each variable to have a mean of zero and a standard deviation of one. To preprocess the data, Z-score normalization was applied to standardize each variable to have a mean of zero and a standard deviation of one. Crucially, the standardization parameters (mean and standard deviation) were computed exclusively from the training set (5968 samples) and then applied to transform both the training and testing sets, thereby preventing data leakage and ensuring that the testing set remains unseen during model training. This approach preserves the independence of the test data and provides an unbiased evaluation of model performance, consistent with best practices in machine learning-based hydrological modeling. Roughly 2% of records were adjusted due to errors or blanks. Figure 2 shows the time series of the Sattarkhan Dam reservoir inflow over a span of 25 years.

### 2.3. Conceptual Framework

In the daily modeling of river discharge for the Sattarkhan Dam region, the input data included inflow delays to reflect the short-term persistence of the hydrological system. To determine the optimal input lag structure for daily river discharge modeling, a systematic lag selection procedure was employed using the partial autocorrelation function (PACF), which isolates the direct correlation between the time series and its lagged values after removing intervening effects. The PACF analysis revealed that the partial autocorrelations for the first three lags were statistically significant at the 95% confidence level, with values of 0.846 (Lag 1), 0.772 (Lag 2), and 0.718 (Lag 3), while partial autocorrelations beyond Lag 3 dropped sharply within the confidence bounds, indicating no significant direct influence of further lags. The 3-day lag was chosen to capture the delayed hydrological response typical for the Sattarkhan Dam region, reflecting the cumulative effects of snowmelt, precipitation events, and soil moisture. This delay period aligns with physical understanding of the system’s short-term persistence. The Sattarkhan catchment does not have a hydrological regime similar to wet regions; it is characterized by high spring snowmelt and low summer flows, while precipitation is generally moderate and evaporation rates are high. By incorporating inflow conditions from the past three days, the model allows for the cumulative and delayed responses of inflows associated with snowmelt, occasional precipitation, and catchment soil moisture conditions. The statistical values used for inflow assessment based on 8952 observations are presented in Table 1.

To evaluate the predictive performance of the developed models, the dataset was partitioned into training (70%) and testing (30%) sets using a chronological split based on the temporal order of observations, preserving the sequential structure of the daily streamflow time series. This approach was selected to maintain the temporal integrity of the data, ensuring that the model is trained on historical observations and tested on future, unseen data, which simulates real-world forecasting conditions where predictions are made for subsequent time periods. The 70–30 split ratio was chosen following common practice in hydrological machine learning studies, providing a sufficiently large training set for model development and hyperparameter optimization, while retaining an independent test set (2980 samples) large enough to yield robust performance estimates. We acknowledge that advanced validation methods such as rolling-origin or expanding-window cross-validation can provide additional robustness by simulating multiple forecast scenarios and reducing dependence on a single split. However, these methods are significantly more computationally expensive, requiring repeated model training and hyperparameter optimization for each time step, which would be prohibitive given the already substantial computational demands of this study, including optimization trials per algorithm and total runtimes. For this study, the chronological split is justified by the need to maintain methodological consistency with prior hydrological modeling studies, the computational constraints of hyperparameter optimization, and the focus on evaluating point, probabilistic, and uncertainty estimates using a clearly defined, independent test period representing the most recent hydrological conditions, while future work will explore rolling-origin validation to assess model stability over time.

The proposed algorithms were tested for their predictive, probabilistic, and uncertainty estimation capabilities using a dataset of 8952 discharge measurements. The dataset was split such that 5968 samples were used for training and 2980 samples were used for testing. This dataset allowed the use of three techniques based on conformal prediction, SplitCP, CV+, and conformal quantile regression, that aim to estimate predictive uncertainty, as well as two machine learning models, NGBoost and PGBM. The hyperparameter of both machine learning models was tuned using the AutoSampler module in Optuna, an automated optimization package for Python V3.13.3.

#### 2.3.1. Uncertainty Quantification Using Conformal Prediction

Conformal prediction (CP) is based on statistical theory, which provides a prediction interval under the assumption that the order of samples does not affect their joint probability distribution [27,28,29]. It makes no assumptions about the data and can be used to quantify uncertainty in predictions with machine learning algorithms. In place of a point estimate (i.e., a single output), CP provides prediction intervals that are guaranteed to contain the true outcome for the majority of test samples with a user-specified probability (i.e., 90% in this study).

Conformal prediction can be applied to any machine learning algorithm, including random forests, gradient boosted trees, support vector machines, and deep learning models [30]. Given their effectiveness in uncertainty estimation, the present study explores the use of three CP methods: split conformal prediction, cross-validation plus, and conformal quantile regression.

Split Conformal Prediction (SplitCP) works by dividing training samples into training and calibration sets [31]. The training set is used to train the machine learning model, whereas the calibration set is used to calculate the nonconformity score using the trained model. The nonconformity score for each calibration data sample is computed using residuals by comparing them with the predicted value. Afterward, the empirical quantile of the nonconformity score is computed to determine the threshold for prediction intervals. Finally, SplitCP uses test samples to create a prediction set comprising all outcomes within the threshold used by the empirical quantile. This approach is computationally efficient and performs well with large sample sizes.

Cross Validation Plus (CV+) method of conformal prediction works by splitting the training samples into multiple folds, and the machine learning model is trained on all folds except one, called the validation dataset [32]. In comparison to Jackknife+, which uses LOOCV, CV+ uses *K*-fold validation, where *K* = 5 was used in this study. The model-building process is carried out on all folds, yielding a total number of models equal to the number of folds used to split the training data. A nonconformity score is then calculated for each sample in the validation fold using its residuals. This score suggests the goodness of predicted values vis a vis the observed data. The nonconformity scores from all folds are combined to obtain a robust estimate of the prediction interval. This combined nonconformity score is then used with each sample in the testing dataset to construct a prediction interval. This approach for smaller-sample-size problems. It is more computationally demanding than SplitCP due to the use of *K*-fold cross-validation.

Conformal quantile regression (CQR) combines quantile regression and conformal prediction to provide prediction intervals [33]. It works well with problems having a limited sample size. CQR first uses quantile regression to produce initial prediction intervals that are sensitive to local uncertainty, and then uses conformal prediction to calibrate these intervals. While calibrating the intervals, it ensures that the proportion of observations lying outside the prediction intervals is within a desired level.

SplitCP was selected as the baseline method due to its computational efficiency and simplicity, requiring only a single model fit and providing valid coverage guarantees under the exchangeability assumption. However, SplitCP sacrifices a portion of the data for calibration, which can lead to wider prediction intervals when data are limited. CV+ addresses this limitation by employing a K-fold cross-validation framework that leverages the entire dataset for both training and calibration, yielding more stable and data-efficient prediction intervals with a finite-sample coverage guarantee of at least 1−2α. Empirical evidence shows that CV+ can reduce interval width by approximately 7% compared to SplitCP while maintaining comparable coverage. The trade-off is computational: CV+ requires training of K models (typically 5 or 10 folds), which was feasible in this study given the moderate dataset size (5968 training samples) and documented optimization times (~20 min per algorithm).

Conformal quantile regression was selected as the third method because it directly addresses heteroscedasticity, a defining characteristic of hydrological time series where prediction uncertainty varies with flow magnitude. Unlike SplitCP and CV+, which produce symmetric intervals around a central prediction, conformal quantile regression estimates conditional quantiles of the target distribution, allowing interval widths to adapt to local data variability. This is particularly advantageous for streamflow forecasting, where higher flows typically exhibit greater uncertainty due to the nonlinearity of rainfall–runoff processes and snowmelt dynamics, with evidence from runoff interval prediction. While conformal quantile regression requires larger sample sizes for stable quantile estimation and is more sensitive to quantile level selection, its ability to produce adaptive, heteroscedastic intervals makes it a valuable complement to SplitCP and CV+. The combined use of these three methods provides a comprehensive assessment: SplitCP offers an efficient baseline, CV+ improves data efficiency and stability, and conformal quantile regression enables adaptive intervals that reflect flow-dependent uncertainty, ensuring that the advantages and limitations of each technique are systematically evaluated for operational streamflow forecasting in semi-arid catchments.

#### 2.3.2. Machine Learning Algorithms

To investigate the potential of three CP-based uncertainty estimation methods, two machine learning algorithms, namely Natural Gradient Boosting (NGBoost) and the probabilistic gradient boosting machine (PGBM), are used in this study. Compared with simple gradient-boosting-based regression tree algorithms (e.g., XGBoost), NGBoost and PGBM provide probabilistic predictions rather than point estimates.

Natural Gradient Boosting (NGBoost) is a machine learning algorithm that provides probabilistic predictions in place of point estimates, as opposed to other gradient boosting algorithms like LightGBM and XGBoost [34]. Unlike models such as XGBoost or LightGBM, which minimize squared error or cross-entropy directly, NGBoost uses the natural gradient to perform gradient boosting in place of the ordinary gradient, improving training stability and efficiency for probabilistic models.

The standard gradient is the direction of steepest ascent in a simple Euclidean space, which can lead to inefficient and unstable updates when dealing with probability distributions. The natural gradient corrects the curvature of the distribution space, yielding a more direct and reliable path to the optimal solution.

To predict a target variable y given input features x, one should assume that y follows a specific probability distribution, PØ(y/x), where *φ* is a set of parameters for the used probability distribution. The loss function is the Negative Log-Likelihood (NLL), also called a Proper Scoring Rule:

(1) $$L\left(Ø,\;y\right)=-logP_{Ø}\left(y/x\right)$$

The natural gradient ($\tilde{g}$) is calculated by scaling the standard gradient (*g*) with the inverse of the Fisher information matrix (*F(φ)*).

(2) $$\tilde{g}={F\left(Ø\right)}^{-1}g$$

The algorithm iteratively builds the predictive model after initializing parameters ${Ø}^{0}$ for all data. For each iteration *I*, it works by computing gradients of the loss with respect to distributional parameters by training a set of base learners, one for each parameter in *φ*. After applying natural gradient correction, it fits a base learner to the corrected gradient. Then it updates the parameters for the next stage using an update rule. For further details of NGBoost, readers are referred to Duan et al. [34] and O’Malley et al. [35].

Probabilistic gradient boosting machine (PGBM) is based on the traditional gradient boosting machine and works by treating leaf weights in decision trees as random variables [36]. Compared to simple gradient boosting machines that provide point estimates, PGBM allows determination of probabilistic estimates using the mean and variance of the test samples. PGBM uses histogram binning to convert continuous features into discrete bins to reduce the computational cost. To start working with PGBM, the mean value of the predicted variable is used to initialize the training process. Then, gradient boosting is used to calculate the gradient and Hessian of the training dataset for a fixed number of iterations. A bootstrapped sample is then used to generate a decision tree with a fixed number of leaves, and the prediction on the entire training dataset is used to obtain a new estimate in place of the mean value. This process is repeated for the required number of iterations.

In gradient boosting-based algorithms, a loss function is optimized by sequentially adding several decision tree-based predictive algorithms to the ensemble. At each iteration *p*, a decision tree $f^{p}\left(x_{i}\right)$ is created so that the update equation of the predicted value for sample *i* can be written as

(3) $${\hat{y}}_{i}^{\left(p\right)}={\hat{y}}_{i}^{\left(p-1\right)}-\rho f^{p}\left(x_{i}\right)$$

where $\hat{y}$ and *ρ* are predicted values of the output variable and the learning rate. While constructing a decision tree in PGBM, a reduction in uncertainty is achieved through the best possible split gain [36]. In case of no split, the node becomes a leaf, and the leaf weight is calculated by:

(4) $$w_{k}=-\frac{\sum_{i=I_{k}}u_{i}}{\sum_{i=I_{k}}v_{i}+\delta }$$

where k∈{0, 1, ……..L}, with L representing the total number of leaves in the tree. Further, δ, *I* and $u_{i},\;v_{i}$ represent the regularization parameter, gradient, and Hessian, respectively. After training a tree, the output for a particular sample in the testing dataset is determined by

(5) $${\hat{y}}_{i}^{\left(p\right)}={\hat{y}}_{i}^{\left(p-1\right)}-\rho w_{k}$$

Finally, to update the mean and variance ($\sigma^{2})$ after each iteration, the following equations are used [36]:

(6) $${mean}_{{\hat{y}}_{i}^{\left(p\right)}}=Expectation\left[{\hat{y}}_{i}^{\left(p\right)}\right]$$

(7) $${\sigma^{2}}_{{\hat{y}}_{i}^{\left(p\right)}}=variance\left[{\hat{y}}_{i}^{\left(p\right)}\right]$$

For further details about PGBM, readers are referred to Sprangers et al. [36].

The use of different machine learning algorithms requires selecting several user-defined parameters (i.e., hyperparameters) that influence their performance with a given dataset. Thus, the need to find optimal hyperparameter values becomes important for comparing various machine learning algorithms. To obtain optimal hyperparameter values for the dataset used in this study, Optuna [37], a Python library for hyperparameter optimization, was used. It includes eleven search methods (i.e., called samplers): Random, Grid, TPE (Tree-structured Parzen Estimator), CMA-ES (covariance matrix adaptation evolution strategy), NSGAII (Nondominated Sorting Genetic Algorithm II), QMC (Quasi Monte Carlo), GP (Gaussian process), partially fixed parameters, NSGAIII, and brute force. Given the high computational cost of using various samplers, the Optuna-based AutoSampler package, which automatically selects the best sampler, was used in this study. The choice of a suitable sampler in AutoSampler depends on the problem, the number of evaluations, the search space, constraints, and the number of objectives [37]. Optuna also uses several pruning techniques to stop unfavorable trials during the hyperparameter optimization process. Pruning helps in stopping trials that are expected not to produce good results, thus saving computational time. For this study, eight pruning approaches (i.e., Median, Nop, patient, percentile, successive halving, hyperband, threshold, and Wilcoxon) as implemented in Optuna were used with the AutoSampler package.

**Figure 3:**
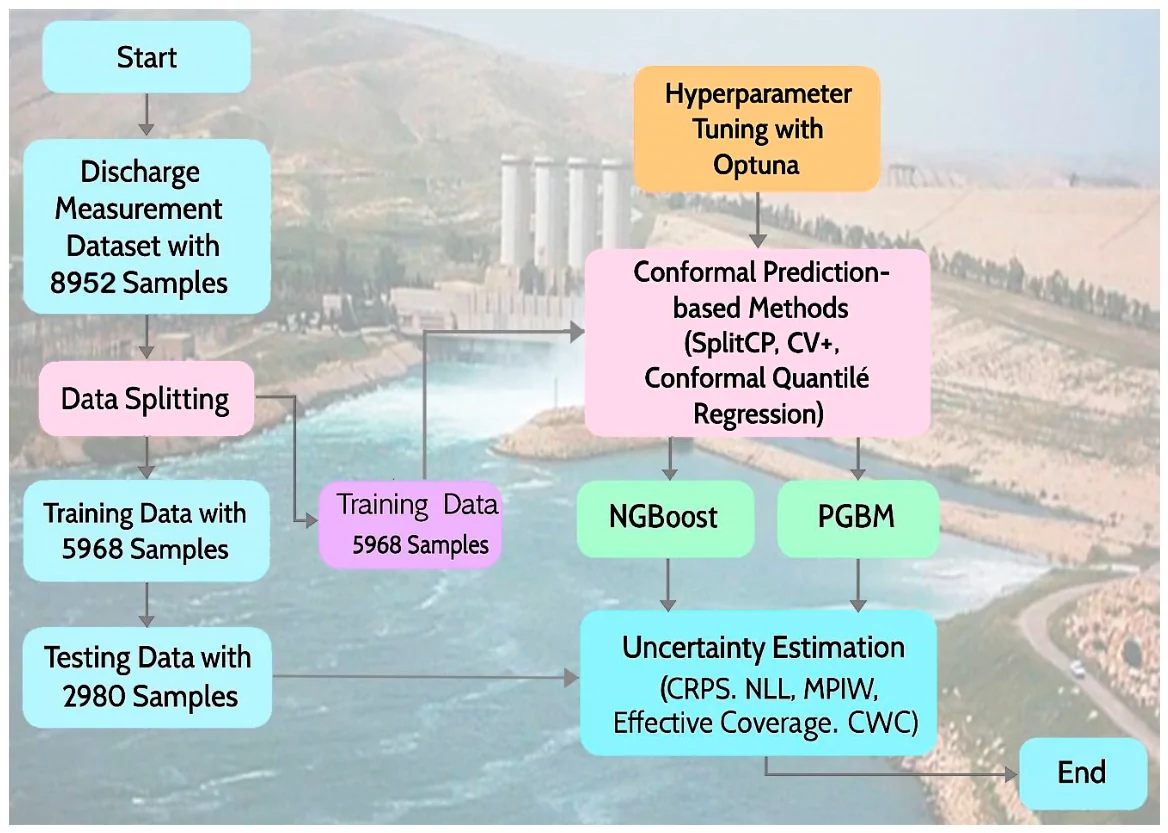
Flowchart of the steps taken to predict the inflow of the Sattarkhan Dam.

For each algorithm (NGBoost and PGBM), a total of 500 optimization trials were conducted, with the Percentile pruner employed to terminate unpromising trials early, thereby reducing computational overhead. The optimization objective was set to minimize the negative log-likelihood (NLL) on a validation subset comprising 20% of the training data, held out for early stopping and performance monitoring. Convergence analysis revealed that both algorithms achieved stable objective values within approximately 350–400 trials, beyond which no significant improvement was observed, confirming that 500 trials were sufficient for identifying near-optimal configurations. The total computational time for the optimization process was approximately 20 min for NGBoost and 18 min for PGBM, with the Percentile pruner effectively terminating approximately 40% of trials before completion, resulting in an estimated 35% reduction in total runtime. The correct optimal depth values are 6 for NGBoost and 8 for PGBM, which were obtained through the Optuna hyperparameter optimization process (500 trials per algorithm, Percentile pruner). These values indicate that NGBoost achieved optimal performance with shallower trees (Depth = 6) compared to PGBM (Depth = 8), reflecting the different regularization and optimization characteristics of the two algorithms. Table 2 provides the optimal values of various hyperparameters with the best pruner using both algorithms. Figure 3 depicts the workflow used to generate daily inflow predictions for the selected dam.

#### 2.3.3. Model Evaluation Metrics

To assess the performance of various machine learning models, three evaluation criteria, namely correlation coefficient (CC), root mean square error (RMSE), and mean absolute error (MAE), were considered. Three scoring criteria, including sharpness, the mean Continuous Ranked Probability Score (CRPS), and the mean of Negative Log-Likelihood (NLL), are used to assess the performance of probabilistic machine learning methods [38]. Sharpness refers to the mean of standard deviation values for each sample in the testing dataset. A lower sharpness value signifies greater uncertainty or less confidence in the predicted result. On the other hand, lower values of CRPS and NLL indicate better, more accurate predictions, leading to a predicted distribution that is closer to the true outcomes.

To assess the quality of conformal prediction-based machine learning models in predicting river discharge, two criteria: mean predicted interval width and effective coverage were used. Effective coverage indicates how often the true value lies within the defined interval. A target coverage of 90% (i.e., confidence level) was used for all CP methods using both machine learning algorithms.

## 3. Results

**Figure 4:**
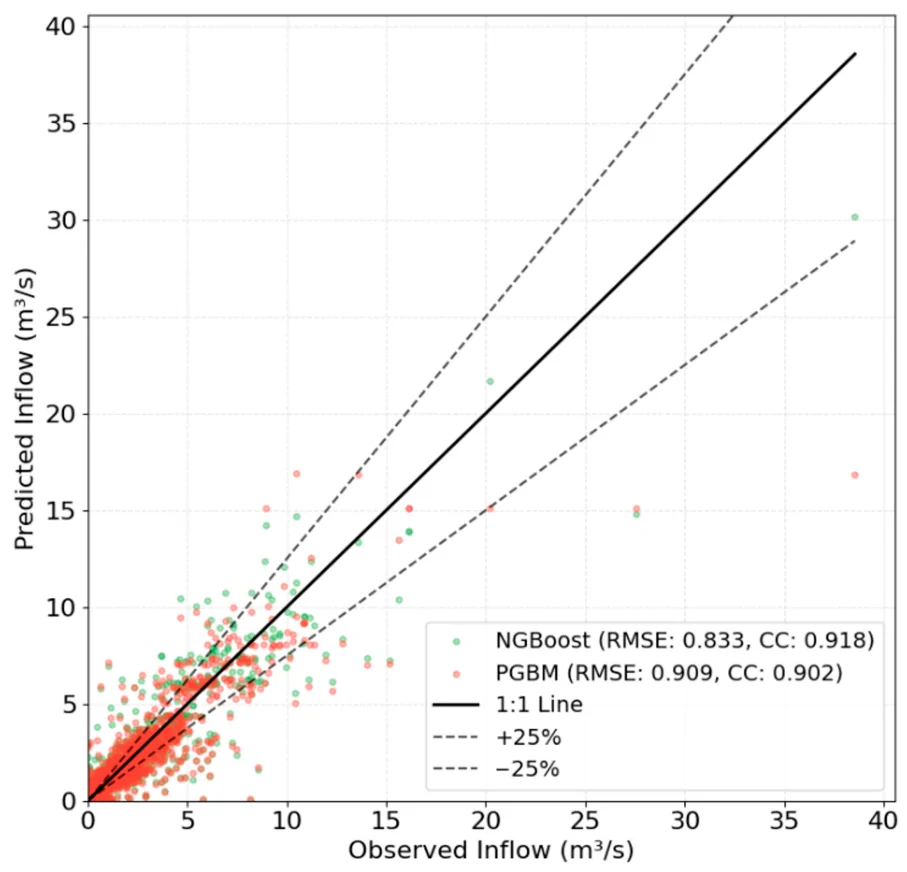
Actual and predicted discharge values with different machine learning algorithms.

To evaluate the performance of machine learning algorithms, CC, RMSE, and MAE values obtained using the optimal combination of hyperparameters are provided in Table 3. Comparison of results in terms of RMSE, CC, and MAE indicates superior performance of NGBoost with this dataset. The graph of actual and predicted river discharge values using the test dataset (Figure 4) indicates that the majority of NGBoost predictions lie closer to the line of perfect agreement, which is supported by lower RMSE and higher CC values (Table 3).

Figure 4 presents the scatter plot of actual versus predicted discharge values, where the predicted values represent the mean predictions (point estimates) generated by each machine learning algorithm (NGBoost and PGBM) before the application of conformal prediction. These mean predictions are obtained directly from the probabilistic models—NGBoost outputs the mean (location parameter) of the conditional distribution $P_{\phi }\left(y|x\right)$ for each input sample, while PGBM provides the expected value (mean) of the predicted discharge based on the leaf weight distributions across the ensemble. The purpose of showing these mean predictions is to evaluate the point estimation accuracy of each model using standard metrics (RMSE, MAE, CC), which serves as a baseline for assessing the added value of uncertainty quantification.

**Figure 5:**
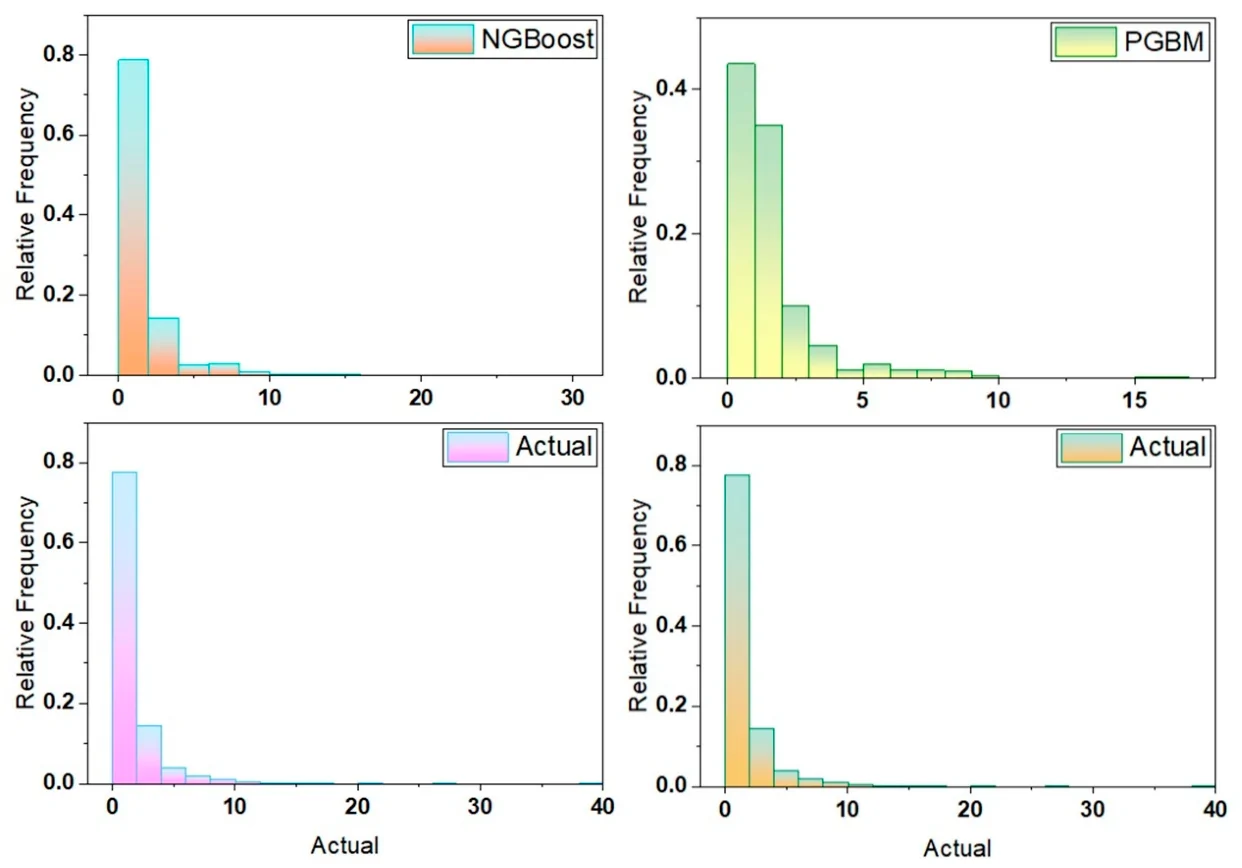
Relative frequency plots of actual and predicted with different machine learning algorithms.

In addition to evaluating the predictive performance of the NGBoost and PGBM models for dam inflow predictions, relative frequency histograms were created to compare the distributions of the predicted and actual values (Figure 5). In the top left and top right of the figure, the NGBoost and PGBM results are illustrated, respectively; both graphics incorporate the histogram of actual inflow values as a base. The incorporation of the histogram into the graphics facilitates visual comparisons of the extent to which empirical distributions are replicated by the predictive models. The bottom row includes reference histograms of the actual inflow data, which serve to reinforce the unimodal shape and spread of observed values.

The NGBoost model strongly represents the empirical distribution of inflow. The predicted results are close to the peak area in the middle and its variations, and numerical validation indicates that NGBoost simulates the mean and variability of the inflow. The minimal deviations in the extremities reflect the model’s constraints in depicting extreme inflow conditions. The combined distribution remains high. The above figure demonstrates the efficacy of the NGBoost model that is designed to probabilistically simulate instead of making point estimations to approximate the whole conditional distribution of the study variable. The PGBM model’s resulting distribution is narrower than the other two models, with slight defects in displaying the peak shape due to the data set. The above observation shows that the PGBM model might be presenting low variability with a higher possibility of leaning towards the mean values of the data set. The resultant shape still has low variability, with potentially higher variations and fluctuations in inflow. The above instances point to the need to assess outcome development in terms of displaying uncertainties in possible predictive models to study the extremities of predictive models. The result demonstrates that the use of the NGBoost model might yield more reliable results due to higher convergence with statistical measures for the dataset representing the dam’s inflow.

**Figure 6:**
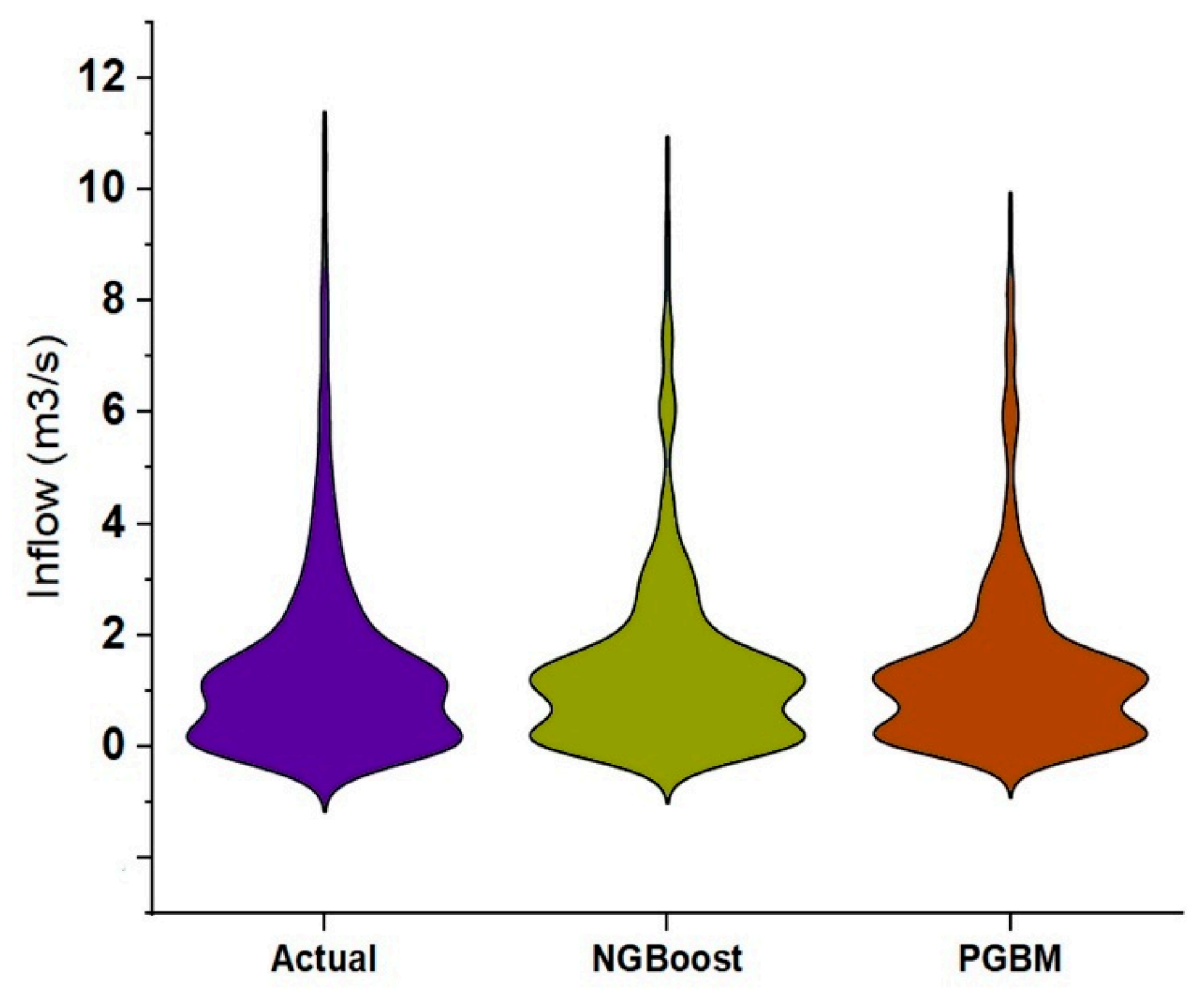
Violin plots of actual and predicted values (predictions made using different machine learning algorithms).

To provide an additional assessment of the distributional accuracy of NGBoost and PGBM in predicting dam inflow, violin plots were created to show the overall density and spread of inflow values for each of the three groups: Actual, NGBoost, and PGBM (Figure 6). Each violin plot shows the overall probability density of inflow (in m^3^/s) on the vertical axis, with the width of each violin determined by relative frequency. This visual representation can provide context for comparing how well each model captures the shape and variability of the observed distribution of inflow values.

The actual inflow distribution is moderately right-skewed and unimodal, and values are concentrated around 2–6 m^3^/s. The NGBoost distribution reflects this pattern well, and NGBoost captures both the central mass of the observed distribution and the tapering tail in both directions. The density curve produced by NGBoost indicates that it captures the variability and uncertainty in the inflow data, including low-probability observations of extremes. The distribution presented by PGBM appears more compact, with a decreased spread and a sharper peak, suggesting that PGBM has underestimated the variance and does not fully capture the tails of the distribution. In summary, the violin plots support the relative frequency histograms: NGBoost provides a better model fit to the full inflow distribution than PGBM, which tends to concentrate predictions around the mean inflow. These differences are important in hydrological prediction, where it is important to simulate both central tendencies and extremes in order to make effective operational decisions. The visual comparison in this study underscores the importance of distribution-aware evaluation metrics and NGBoost’s advantage in probabilistic inflow prediction.

In addition to point estimates, both machine learning algorithms were evaluated on their probabilistic predictions for each test sample, reported as mean and standard deviation values. Along with these two values, both CRPS and NLL values were also computed. Table 4 provides the sharpness, mean CRPS, and mean NLL values from both machine learning algorithms.

The sharpness metric, defined as the mean width of the predictive distribution, indicates that PGBM produces considerably narrower prediction intervals (sharpness = 0.02) compared to NGBoost (sharpness = 0.49). However, sharpness alone is insufficient for evaluating probabilistic forecast quality, as it must be interpreted jointly with calibration (coverage) and proper scoring rules. The extremely high negative log-likelihood (NLL = 909.21) for PGBM, despite its low sharpness, reveals a critical issue: the model is severely overconfident, generating unrealistically narrow predictive distributions that fail to capture the actual variability and uncertainty in the observed discharge values. This behavior is characteristic of underfitting or misspecification, where the model underestimates the inherent noise in the hydrological system. In contrast, NGBoost’s wider predictive distributions (sharpness = 0.49) are accompanied by a substantially lower NLL (1.44) and lower CRPS (0.29 versus 0.38 for PGBM), indicating that NGBoost achieves a better balance between sharpness and calibration; its predictions are appropriately uncertain, assigning higher probabilistic density to the true observed values. This is further supported by the conformal prediction results, where NGBoost with CV+ achieved effective coverage closest to the nominal target, confirming that its wider intervals are well-calibrated. Therefore, the combination of sharpness, CRPS, and NLL demonstrates that NGBoost produces probabilistically superior and more reliable forecasts, while PGBM’s overly narrow intervals are misleading and practically unusable for risk-aware decision-making in water resource management.

**Figure 7:**
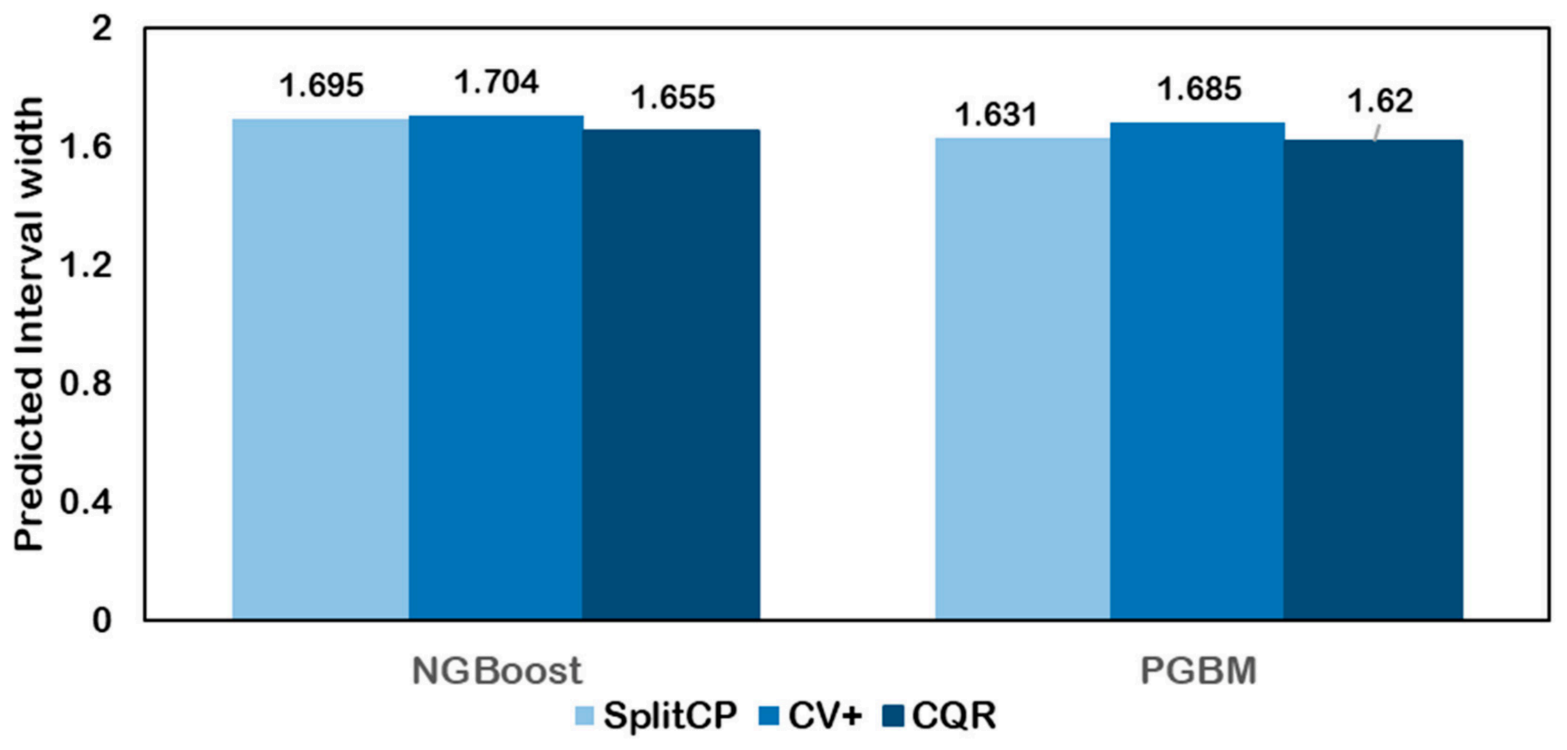
Predicted interval width using different CP methods with models.

**Figure 8:**
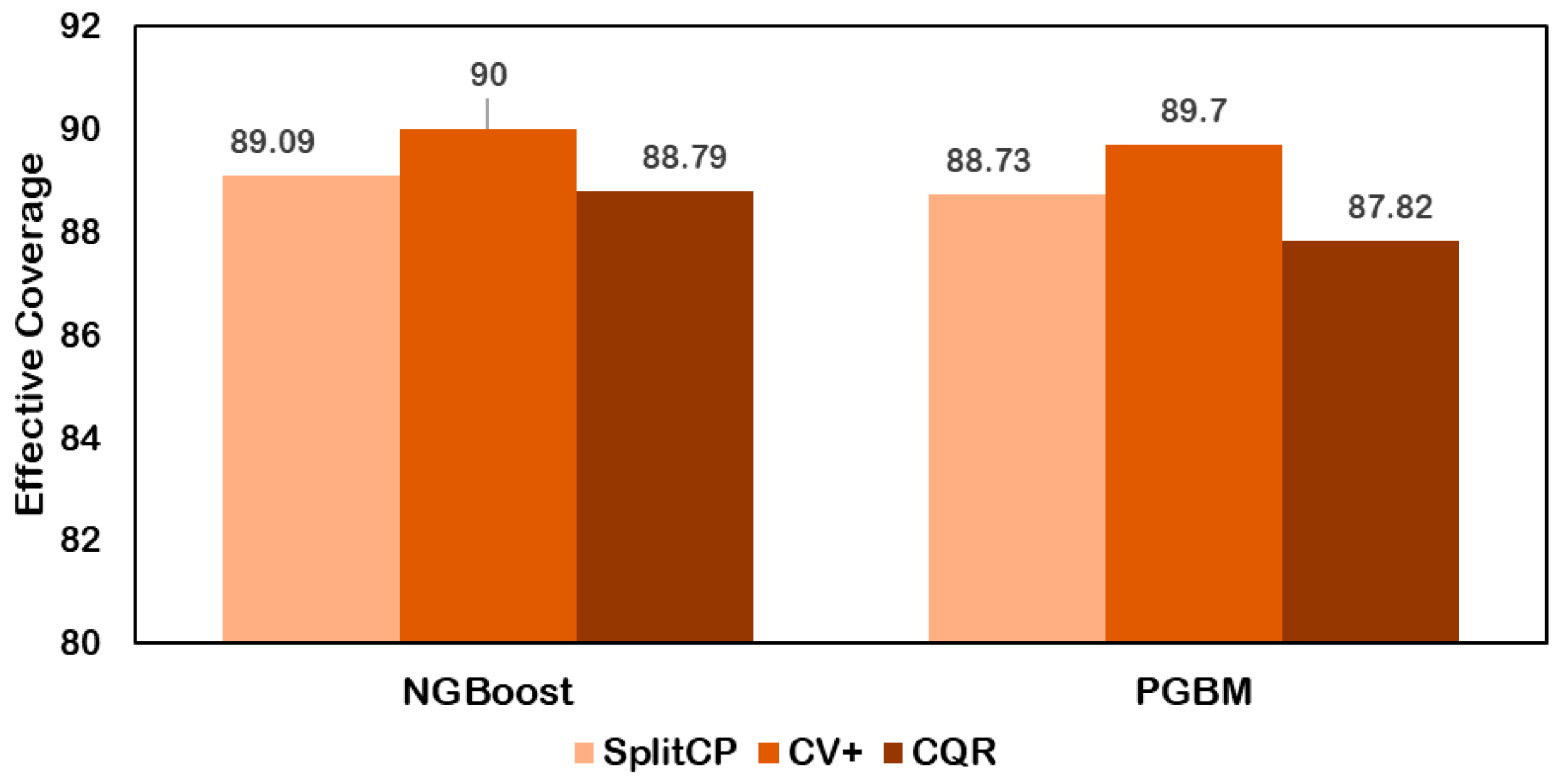
Effective coverage using different CP methods with models.

Results of conformal prediction-based uncertainty estimation in terms of mean predicted interval width and effective coverage are plotted in Figure 7 and Figure 8. Figure 7 depicts a graph representing the mean predicted interval width by different conformal prediction approaches using both machine learning algorithms. A smaller mean interval width indicates precise predictions leading to high confidence, whereas a higher value indicates greater uncertainty in the predictions.

**Figure 9:**
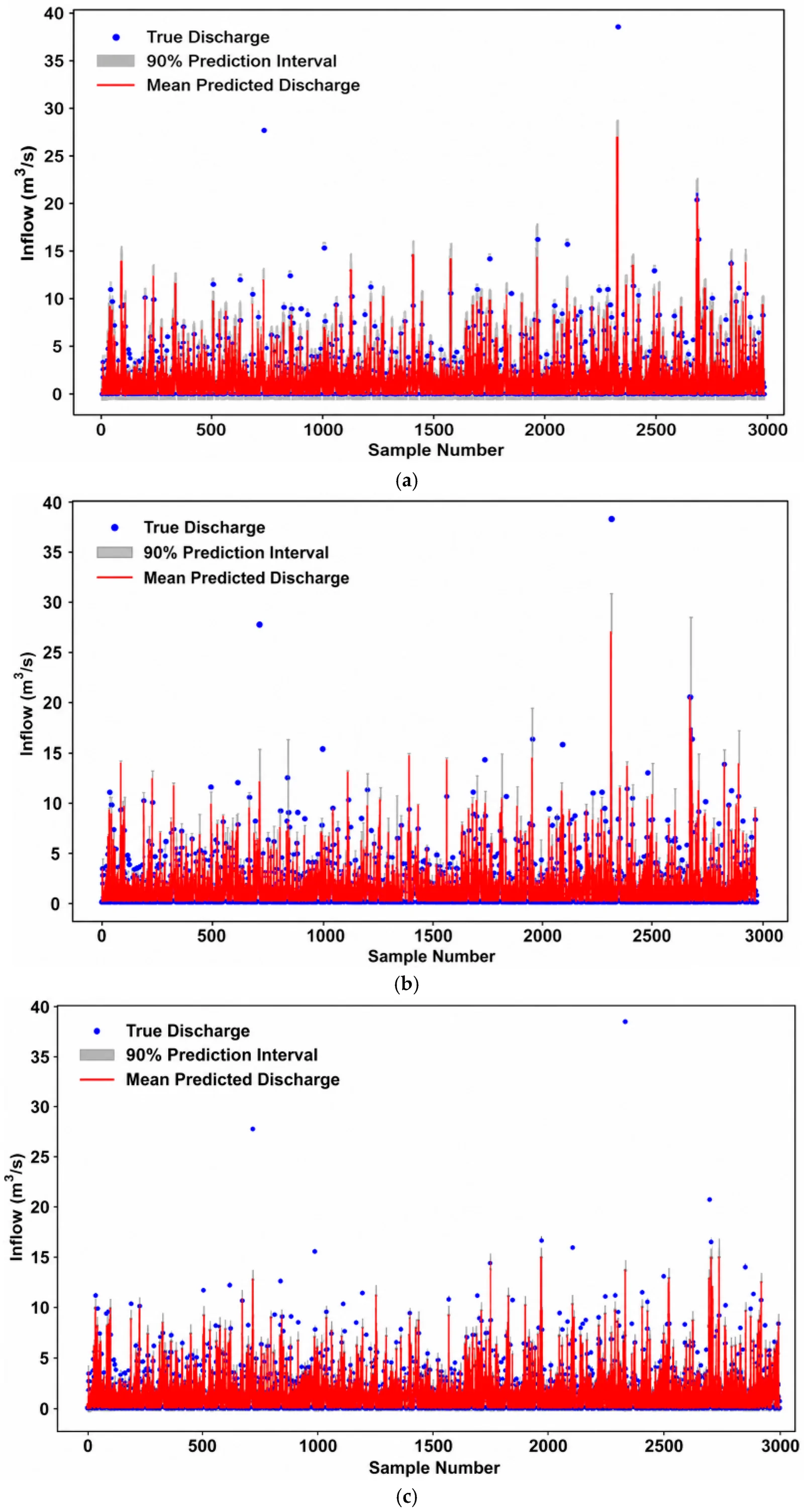
(**a**) Plot of predicted interval using NGboost with SplitCP. (**b**) Plot of predicted interval using NGBoost with CV+. (**c**) Plot of predicted interval using NGBoost with CQR.

Results from Figure 7 indicate better performance by the SplitCP approach in terms of predicted interval width with both ML algorithms. On the other hand, Figure 8 indicates that, except for the CV+ approach with NGBoost, no other method satisfies the 90% coverage criteria with both ML models. Thus, suggesting the effectiveness of the CV+ conformal prediction approach in capturing uncertainty. Considering the superior performance of NGBoost, the output, both in terms of mean predicted values and sample-wise predicted intervals using 90% target coverage, is also plotted (Figure 9).

Comparison of Figure 9a–c suggests that the SplitCP-based CP approach yields a narrow-predicted interval compared to both the CV+ and CQR approaches. The prediction interval plot (Figure 7) with all three approaches also suggests that the target coverage covered most of the test samples, except for a few large dam inflow values. Figure 9 also shows that the mean predicted values are quite close to the true values, indicating better predictive accuracy for the NGBoost model across all three CP approaches.

In combination, probabilistic machine learning and conformal prediction improve river discharge modeling by addressing epistemic uncertainty, arising from limited knowledge of the system, and uncertainty due to inherent variability. As a result, these improved modeling methods can inform dam operations, flood-warning systems, and irrigation scheduling decisions. The uncertainty bands generated from these methods will aid the authorities responsible for the Sattarkhan Dam in optimizing reservoir releases under various uncertainty scenarios and in planning for hydrological extremes. Therefore, estimating uncertainty using advanced AI-based methods is critical for resilient and sustainable water management in river basins with dam regulation.

To assess whether the observed performance differences between NGBoost and PGBM were statistically significant, we applied the paired *t*-test to the absolute errors obtained from the 2980 test samples for both models. This test evaluates the null hypothesis that the mean difference between paired error values is zero; rejection of this hypothesis indicates a statistically significant difference between the two models. The paired *t*-test is appropriate here because the predictions from both models are generated on the same test samples, creating paired observations. The test yielded a t-statistic of −8.57 with a *p*-value of 2.8 × 10^−18^ (*p* < 0.001), leading to rejection of the null hypothesis and confirming that NGBoost’s superior point forecast performance is statistically significant. The negative t-statistic indicates that the mean absolute error for NGBoost is significantly lower than that for PGBM, with the distribution of paired differences showing a consistent improvement across the majority of test samples. Additionally, for probabilistic forecast evaluation, we applied the Diebold–Mariano test to compare the CRPS values of the two models, which also confirmed a statistically significant difference favoring NGBoost (*p* < 0.05). These findings substantiate the reliability of NGBoost’s superior predictive accuracy and probabilistic calibration, demonstrating that the observed improvements are not attributable to random chance.

The statistical characteristics presented in Table 5 provide valuable insight into model performance across the flow distribution. The maximum values reveal a critical limitation of PGBM: while the actual maximum discharge reached 38.560 MCM, PGBM only predicted 16.919 MCM, a severe underestimation of peak flows by approximately 56%. In contrast, NGBoost predicted a maximum of 35.178 MCM, much closer to the observed peak (only 8.8% underestimation), demonstrating NGBoost’s superior ability to capture extreme events. Furthermore, the standard deviation of NGBoost predictions (1.923) is closer to the actual standard deviation (2.094) compared to PGBM (1.801), indicating that NGBoost better preserves the overall variability of the time series, including the spread associated with both high and low flows. The minimum values also show that NGBoost (0.066) is closer to the observed minimum (0) than PGBM (0.002), though both approaches exhibit slight overestimation at the lower tail.

The statistical characteristics presented in Table 5 provide direct evidence of model performance across the full flow distribution, including extremes. While the actual maximum discharge reached 38.560 MCM, PGBM severely underestimated peak flows by approximately 56% (predicting only 16.919 MCM), whereas NGBoost predicted a maximum of 35.178 MCM, an underestimation of only 8.8%, demonstrating superior ability to capture flood events. Additionally, NGBoost’s standard deviation (1.923) more closely matches the actual variability (2.094) compared to PGBM (1.801), indicating better preservation of the overall flow distribution.

The high CC for PGBM (0.902), despite its significant underestimation of peak flows, reflects the scale-invariant nature of the correlation coefficient, which measures linear relationship strength rather than bias or error magnitude. As shown in Table 5, PGBM underestimated the actual maximum discharge (38.560 MCM) by approximately 56% (predicting only 16.919 MCM), while NGBoost predicted 35.178 MCM (only 8.8% underestimation). However, PGBM’s predictions maintain a strong linear relationship with observations across low to moderate flows, resulting in a high CC despite poor extreme event performance. This is why RMSE and MAE provide complementary information: RMSE (0.833 vs. 0.909) is more sensitive to the large errors associated with peak underestimation due to the squaring of residuals, while MAE (0.375 vs. 0.388) captures the average absolute error. The combination of these metrics reveals that NGBoost not only captures the overall trend but also maintains accuracy across the full flow range, including extremes, whereas PGBM’s high CC masks its critical limitation in forecasting flood events.

## 4. Discussion

Expanding on the topic of uncertainty quantification, this work builds on the application of conformal prediction methods, specifically SplitCP, CV+, and conformal quintile regression, using the probabilistic machine learning models NGBoost and PGBM. These combinations provide a principled way to produce calibrated prediction intervals, providing reliability in streamflow forecasts. Specifically, NGBoost’s natural gradient boosting and PGBM’s probabilistic tree-based learning were combined with the conformal approaches to develop calibrated prediction capacity. By doing so, conformal approaches will produce robust distribution-free uncertainty bounds that adapt to variability in the data. This work is a natural extension of previously demonstrated Bayesian and ensemble methods, which advocate hybrid strategies for hydrological forecasting.

Among the three conformal prediction techniques evaluated, CV+ with NGBoost achieved the most reliable coverage, closest to the nominal target, due to two factors. First, CV+’s cross-validation framework leverages the entire training dataset for both model fitting and calibration, averaging conformity scores across multiple training folds to reduce the influence of any particular training–validation split. This yields more stable prediction intervals compared to SplitCP, which uses a single calibration split and can exhibit high variability depending on the random partition. Second, CV+ demonstrated remarkable robustness to the violation of exchangeability, the fundamental assumption underlying conformal prediction, which is inherently violated by the temporal dependence in daily streamflow data (confirmed by PACF showing significant lags at 1–3 days with correlations of 0.846, 0.772, and 0.718). While SplitCP and conformal quantile regression showed more pronounced degradation, CV+ maintained coverage close to nominal levels, likely because its cross-validation framework implicitly averages over different temporal partitions, smoothing out regime-specific anomalies. This finding aligns with recent advances in time-adaptive conformal prediction and suggests that even standard CV+ offers pragmatic improvements for operational hydrological forecasting where strict exchangeability cannot be guaranteed.

The present findings both corroborate and extend previous work in probabilistic hydrological forecasting. Ghobadi and Kang [39] demonstrated that Bayesian deep learning (BLSTM) achieved superior reliability and sharpness across U.S. catchments through variational inference, while results show that tree-based probabilistic models with natural gradient optimization can achieve comparable benefits without the computational demands of deep learning architectures, requiring only 20 min for hyperparameter optimization on a standard workstation versus days of GPU-accelerated training for BLSTM. Roy et al. [40] combined the HBV hydrological model with Bayesian Particle Filtering and Random Forest to reduce prediction uncertainty, demonstrating that hybrid process-based and machine learning approaches improve accuracy. This study complements this by showing that purely data-driven models, when properly regularized through natural gradient optimization and conformal prediction, can also produce reliable uncertainty estimates without requiring a physically based model component, a particularly valuable finding for data-scarce regions where process-based models may be difficult to calibrate. Le et al. [41] used SHAP analysis to identify key predictors for bedload prediction, while our PACF-based lag selection (identifying Lags 1–3 with strong partial autocorrelations) effectively substituted for post-hoc feature importance analysis, demonstrating that systematic lag selection can be equally informative when temporal dependencies dominate. Vinokić et al. [42] found that TKAN exceeded LSTM but slightly lagged behind TCN for streamflow forecasting, with uncertainty levels of 35.02% for 3-day horizons, whereas this study’s emphasis on conformal prediction provides a more complete picture of forecast reliability through explicit uncertainty bounds rather than point predictions alone.

NGBoost’s key advantage lies in its use of natural gradient descent, which accounts for the Riemannian geometry of the probabilistic parameter space by rescaling the gradient using the Fisher information matrix [34]. Unlike ordinary gradients, which can be highly unsuitable for learning multi-parameter probability distributions (such as the Normal distribution), natural gradients lead to more stable and efficient training dynamics and a better fit. This enables NGBoost to jointly optimize location and scale parameters, capturing heteroscedasticity, a critical feature for hydrological predictions where uncertainty varies with flow magnitude. Furthermore, NGBoost’s modular design allows it to be used with any base learner, any family of distributions with continuous parameters, and any scoring rule (e.g., CRPS), offering flexibility that deterministic models like XGBoost and Random Forest lack [34]. A recent study comparing boosting algorithms for streamflow simulation in the Lower Godavari Basin, India, found that NGBoost achieved the highest Kling–Gupta Efficiency (KGE) values (0.95 training, 0.95 testing), outperforming XGBoost (0.91, 0.90) and other boosting methods, confirming NGBoost’s superior performance in hydrological contexts [43]. Additionally, NGBoost has demonstrated particularly strong performance on smaller datasets, making it well-suited for applications where data may be limited, a common challenge in hydrological modeling.

Application of NGBoost to hydrological uncertainty quantification demonstrated that natural gradient-based probabilistic boosting can produce well-calibrated forecasts with computational efficiency comparable to traditional gradient boosting, opening new avenues for uncertainty-aware modeling without expensive ensemble or Bayesian methods. The rigorous empirical evaluation of three conformal prediction variants (SplitCP, CV+, and conformal quantile regression) for streamflow forecasting demonstrates that CV+ offers a pragmatic, computationally efficient approach for generating prediction intervals that maintain valid coverage even with temporal dependence, a critical requirement for operational applications. Furthermore, the findings provide valuable model selection guidance for semi-arid catchments, a hydrological regime common across the Middle East, Central Asia, and parts of the southwestern United States, showing that models with less aggressive regularization (like NGBoost) may be preferred for catchments with strong seasonal patterns, while heavily regularized models may be better suited to noisier or flashier systems. The computational feasibility makes the framework operationally viable for water management agencies with limited resources, enabling real-time forecasting for reservoir operations, flood warning systems, and irrigation scheduling.

The Sattarkhan Dam was selected as a single-site testbed for developing the proposed probabilistic forecasting framework, chosen for its 26-year high-quality discharge record, its hydrologically complex semi-arid regime that provides a rigorous test for uncertainty quantification methods, and its operational importance for water supply and irrigation. However, the methodological framework itself, encompassing systematic lag selection, automated hyperparameter optimization, natural gradient-based probabilistic boosting, and conformal prediction, is algorithmically transferable and is presented as a template for future applications. The documented optimization protocols, convergence behavior, and evaluation workflows provide benchmarks for researchers adapting this framework to other catchments, and systematic multi-catchment validation is explicitly identified as a priority for future work.

While it is acknowledged that meteorological factors such as precipitation, temperature, and evaporation are fundamental drivers of hydrological processes, the present study employs antecedent inflow (Lags 1–3) as the primary input, a decision justified by both empirical evidence and the specific characteristics of the Sattarkhan catchment. First, the strong partial autocorrelations observed (0.846, 0.772, and 0.718 for Lags 1–3) indicate that streamflow persistence dominates daily variability, with antecedent runoff accounting for 65.9–84.7% of predictive importance in data-driven hydrological models according to recent studies [44,45]. Second, the spatial sparsity of meteorological stations and the coarse resolution of gridded datasets in this semi-arid region introduce significant biases that can degrade model performance; previous research has demonstrated that adding such low-quality meteorological inputs often provides marginal improvement or even degrades accuracy compared to models using only historical runoff data. Third, the Sattarkhan catchment’s semi-arid regime with high evaporation rates and snowmelt-dominated spring flows means that antecedent inflow inherently integrates the cumulative effects of all upstream processes, including precipitation, snowmelt, soil moisture dynamics, and evaporation, making it a reliable surrogate for short-term (1-day ahead) forecasting. This is further supported by the moderate performance (R^2^ = 0.83 calibration, 0.51 validation) of physically based rainfall–runoff models (IHACRES) applied to the same catchment, suggesting that even with explicit meteorological inputs, the rainfall–runoff relationship in this region remains difficult to capture [46].

The selection of NGBoost and PGBM as the two probabilistic machine learning algorithms in this study is justified by their status as complementary state-of-the-art approaches to probabilistic gradient boosting that have recently emerged as reference benchmarks in probabilistic regression literature [47]. While conventional point-estimation models such as Random Forest, XGBoost, ARIMA, and SWAT provide deterministic predictions without explicit uncertainty quantification, the probabilistic nature of NGBoost and PGBM enables a direct comparison of uncertainty estimation capabilities, a central objective of this study. NGBoost employs natural gradient descent to jointly optimize multiple parameters of a specified probability distribution, accounting for the Riemannian geometry of the probabilistic parameter space and enabling stable convergence when capturing heteroscedasticity in hydrological predictions. In contrast, PGBM achieves probabilistic estimates through stochastic tree leaf weights based on sample statistics, offering distinct advantages such as the ability to select an output distribution after training without retraining and training times up to several orders of magnitude faster than NGBoost on larger datasets. Evidence from benchmark regression datasets demonstrates that NGBoost and PGBM frequently outperform other probabilistic methods and serve as standard reference baselines for evaluating new approaches [48]. Given the computational demands of hyperparameter optimization and the need for in-depth analysis of probabilistic outputs, including sharpness, CRPS, NLL, and conformal prediction coverage, focusing on these two leading probabilistic boosting methods provides a rigorous foundation for advancing uncertainty quantification in hydrological modeling, while acknowledging that systematic comparison with conventional models (XGBoost, Random Forest, SWAT, ARIMA, LSTM) across multiple catchments is identified as a priority for future work.

This research has several limitations that should be acknowledged. The study relies solely on a single dataset from the Sattarkhan Dam, which limits the generalizability of these findings to other hydrological contexts and regions such as humid, snow-dominated, or large river systems. The analysis is restricted to two probabilistic machine learning models (NGBoost and PGBM), and the findings may not extend to other probabilistic or non-probabilistic approaches. Additionally, the conformal prediction methods employed assume data exchangeability, which may not hold for real-world time-dependent flow processes, potentially affecting coverage guarantees. The exclusion of meteorological variables (precipitation, temperature, evaporation) limits the model’s ability to capture long-term hydrological responses, particularly under changing climatic conditions. Furthermore, the computational burden of hyperparameter tuning using Optuna, while feasible for this study, may present challenges for operational settings with strict real-time constraints. Finally, extreme events, including flood peaks and drought periods, were not explicitly evaluated using event-specific metrics, which is essential for operational early warning and reservoir management applications. These limitations provide direction for future research, including systematic multi-catchment evaluations, incorporation of meteorological inputs through hybrid modeling, development of time-adaptive conformal methods, and comprehensive extreme-event validation.

A key methodological limitation of this study concerns the conformal prediction methods employed, which assume data exchangeability, a condition fundamentally violated by daily streamflow time series that exhibit strong temporal dependence, as confirmed by the PACF analysis showing significant partial autocorrelations at Lags 1–3 (0.846, 0.772, and 0.718). Temporal dependence causes conformity scores to become correlated across time, meaning that empirical coverage on the calibration set may not accurately reflect coverage on future test points, potentially resulting in either under-coverage (intervals that are too narrow) or over-conservatism (excessively wide intervals), both of which undermine the reliability of uncertainty estimates for operational decision-making. Recent theoretical work has shown that split conformal prediction can still perform effectively in time-series settings when predictors utilize past observations, but coverage loss depends on the extent to which temporal dependence creates violations of exchangeability. While CV+ demonstrated greater robustness to this violation due to its cross-validation framework implicitly averaging over different temporal partitions, the exchangeability assumption remains a fundamental constraint for all three methods. To address this limitation in future research, time-adaptive conformal methods such as Adaptive Conformal Inference (ACI), Conformal PID Control, and autocorrelated multi-step conformal prediction (AcMCP), which preserve the dependence structure among nonconformity scores at the calibration stage, have been proven to guarantee asymptotic marginal coverage for time-series predictions and should be explored for operational hydrological forecasting.

A critical limitation of this study is its validation on a single semi-arid catchment, which constrains generalizability to humid tropical, snow-dominated boreal, large river, and ungauged systems where performance may differ due to variations in lag structure, memory effects, spatial heterogeneity, and data availability. To address this, a comprehensive multi-basin verification plan is proposed for future research, encompassing: (i) selection of 10–15 representative catchments across diverse climates and scales; (ii) standardized application of the framework with catchment-specific adaptations to input features, hyperparameters, and distributional assumptions; (iii) evaluation using point, probabilistic, conformal, and extreme-event metrics; (iv) sensitivity analyses to identify key performance drivers; (v) exploration of transfer learning for data-scarce basins; and (vi) operational validation with water management agencies. This structured approach will establish generalizable guidelines for model selection and uncertainty quantification across global hydrological regimes, supporting the transition from deterministic to risk-based water resource management.

## 5. Conclusions

This study investigated probabilistic prediction and uncertainty estimation for river discharge modeling using three conformal prediction methods (SplitCP, CV+, and conformal quantile regression) combined with two probabilistic machine learning algorithms (NGBoost and PGBM) in the semi-arid Sattarkhan Dam catchment, Iran. The analysis demonstrates that NGBoost consistently outperforms PGBM across all evaluation metrics, achieving superior point estimation (RMSE: 0.833 vs. 0.909 m^3^/s; CC: 0.918 vs. 0.902; MAE: 0.375 vs. 0.388) and significantly better probabilistic forecasts (CRPS: 0.29 vs. 0.38; NLL: 1.44 vs. 909.21), with statistical significance confirmed through paired *t*-test (*p* < 0.001) and the Diebold–Mariano test (*p* < 0.05). Notably, NGBoost demonstrated superior ability to capture extreme events, predicting maximum discharges of 35.178 MCM compared to the actual 38.560 MCM, while PGBM severely underestimated peaks at only 16.919 MCM (56% underestimation). CV+ with NGBoost emerged as the most effective uncertainty estimation method, achieving coverage closest to the nominal target due to its cross-validation framework that leverages the entire dataset for both training and calibration, providing robustness to temporal dependence in streamflow data. The methodological framework developed herein, comprising PACF-based lag selection, Optuna-driven hyperparameter optimization, probabilistic gradient boosting, and conformal prediction, provides a transferable template that can be adapted to different catchments by adjusting input features, hyperparameters, and distributional assumptions. The broader implications extend to operational water resource management, where well-calibrated prediction intervals enable risk-informed decision-making in reservoir operations, flood warning systems, irrigation scheduling, and hydropower generation, supporting adaptive strategies under changing climatic conditions. In summary, combining natural gradient-based probabilistic boosting with CV+ conformal prediction provides a robust, computationally feasible, and transferable framework for generating reliable streamflow predictions with well-calibrated uncertainty intervals, supporting the transition from deterministic to risk-based water resource management in semi-arid catchments and beyond.

Future work can expand the evaluation of hydrological datasets by considering multiple catchments and climates, including humid tropical, snow-dominated boreal, and large regulated river systems, to increase the robustness and generalizability of findings, while also incorporating meteorological variables such as precipitation, temperature, and potential evapotranspiration either as direct inputs to machine learning models or through hybrid approaches coupling conceptual hydrological models (e.g., IHACRES, SWAT, GR4J) with natural gradient boosting, which have been shown to significantly enhance prediction accuracy by combining physical interpretability with data-driven flexibility. Future work can better explicate the utility of additional probabilistic machine learning models and deep learning architectures, such as Bayesian neural networks, variational autoencoders, and transformer-based time-series models, to facilitate improved predictive and uncertainty estimates across diverse hydrological regimes. Future work should more carefully consider the use of conformal prediction methods for time-series or spatially correlated hydrological data, exploring time-adaptive variants such as online conformal prediction, block-wise conformal methods, or distributional conformal prediction that explicitly account for nonstationarity, temporal dependence, and changing climate conditions. Finally, future work should examine real-time methods and computational optimization for operational use in hydrological prediction models, exploring transfer learning to reduce retraining costs, early stopping strategies, and GPU acceleration to enable real-time forecasting for reservoir operations, flood warning systems, and irrigation scheduling. Systematic multi-catchment studies are particularly needed to establish generalizable guidelines for model selection, hyperparameter configuration, input variable selection (including the optimal balance between antecedent flow and meteorological inputs), and conformal prediction adaptation across the diversity of global hydrological regimes, ultimately supporting the transition from deterministic to risk-based water resource management through reliable, well-calibrated uncertainty intervals.

## Figures and Tables

**Table 1:** Statistical characteristics of the parameters used for the modeling.

Statistic	Inflow (m^3^/s)
Minimum	0.000
Maximum	43.420
Range	43.420
1st Quartile	0.120
Median	1.080
3rd Quartile	1.770
Mean	1.478
Variance (*n* − 1)	4.656
Standard deviation (*n* − 1)	2.158
Skewness (Pearson)	5.752

**Table 2:** Optimal values of various hyperparameters used during the training of different algorithms.

Hyperparameter	Description	NGBoost	PGBM
Pruner	Pruning strategy for terminating unpromising trials	Percentile	Percentile
Learning rate	Step size shrinkage for updating predictions	0.01	0.01
Iterations	Number of boosting rounds	500	300
Depth	Maximum depth of each tree	6	8
Sub sample	Fraction of training data used per boosting iteration	1.0	—
Colsample by Level	Fraction of features randomly sampled at each tree level	0.7	—
Natural Gradient	Enables natural gradient for parameter updates	True	—
Bagging fraction	Fraction of training data sampled per iteration	—	0.7
Feature fraction	Fraction of features randomly selected per iteration	—	1.0
Min Split Gain	Minimum loss reduction required for a new split	—	0.1
Tree correlation	Controls dependence between successive trees	—	0.2
Minimum data in leaf	Minimum samples required in a leaf node	—	3
Max bin	Maximum bins for discretizing continuous features	—	256
No. of leaves	Maximum leaf nodes allowed per tree	—	31
Lambda	L2 regularization parameter to control overfitting	—	5

**Table 3:** Statistical parameters using different machine learning algorithms with optimal hyperparameters.

	NGBoost	PGBM
RMSE (m^3^/s)	0.833	0.909
CC	0.918	0.902
MAE	0.375	0.388

**Table 4:** Evaluation matrix for probabilistic prediction using machine learning algorithms.

Algorithm	Sharpness	Mean CRPS	Mean NLL
NGBoost	0.49	0.29	1.44
PGBM	0.02	0.38	909.21

**Table 5:** Statistical characteristics of model predictions compared to actual.

Statistical Criteria	Minimum (MCM)	Average (MCM)	Maximum (MCM)	Standard Deviation (SD)
Actual	0	1.510	38.560	2.094
NGBoost	0.066	1.507	35.178	1.923
PGBM	0.002	1.497	16.919	1.801

## Data Availability

The data used in this study were obtained from the Regional Water Company of East Azerbaijan Province, Iran. Data can be sent upon request and with official permission from this company.
